# Importance of hereditary and selected environmental risk factors in the etiology of inflammatory breast cancer: a case-comparison study

**DOI:** 10.1186/s12885-016-2369-z

**Published:** 2016-05-26

**Authors:** Roxana Moslehi, Elizabeth Freedman, Nur Zeinomar, Carmela Veneroso, Paul H. Levine

**Affiliations:** Department of Epidemiology and Biostatistics, School of Public Health, University at Albany, Albany, NY 12144 USA; Cancer Research Center, University at Albany, Room 208, 1 Discovery Drive, Albany, NY 12144 USA; Department of Epidemiology and Biostatistics, School of Public Health and Health Services, The George Washington University, Washington, DC 20037 USA; Department of Epidemiology, College of Public Health, University of Nebraska, Omaha, NE 68198 USA

**Keywords:** Inflammatory breast cancer, IBC, Heredity, Case-comparison study, Family history of breast cancer, Breast cancer risk factors, Multiplex IBC families

## Abstract

**Background:**

To assess the importance of heredity in the etiology of inflammatory breast cancer (IBC), we compared IBC patients to several carefully chosen comparison groups with respect to the prevalence of first-degree family history of breast cancer.

**Methods:**

IBC cases (*n* = 141) were compared to non-inflammatory breast cancer cases (*n* = 178) ascertained through George Washington University (GWU) with respect to the prevalence of first-degree family history of breast cancer and selected environmental/lifestyle risk factors for breast cancer. Similar comparisons were conducted with subjects from three case–control studies: breast cancer cases (*n* = 1145) and unaffected controls (*n* = 1142) from the Cancer Genetic Markers of Susceptibility (CGEMS) study, breast cancer cases (*n* = 465) and controls (*n* = 9317) from the Women’s Health Initiative (WHI) study, and ovarian cancer cases (*n* = 260) and controls (*n* = 331) from a study by University of Toronto (UT).

**Results:**

The frequency of first-degree breast cancer family history among IBC cases was 17.0 % compared to 24.4 % for GWU breast cancer cases, 23.9 % and 17.9 % for CGEMS breast cancer cases and controls, respectively, 16.9 % and 12.6 % for WHI breast cancer cases and controls, respectively, and 24.2 % and 11.2 % for UT ovarian cancer cases and controls, respectively.

IBC cases had a significantly lower prevalence of parous women than WHI breast cancer cases (OR = 0.46, 95 % CI:0.27–0.81) and controls (OR = 0.31, 95 % CI:0.20–0.49). Oral contraceptive use was significantly higher among IBC cases compared to WHI breast cancer cases (OR = 7.77, 95 % CI:4.82–12.59) and controls (OR = 8.14, 95 % CI:5.28–12.61). IBC cases had a significantly higher frequency of regular alcohol consumption (≥1 drink per day) compared to WHI controls (OR = 1.84, 95 % CI:1.20–2.82) and UT controls (OR = 1.86, 95 % CI:1.07–3.22) and higher (statistically non-significant) prevalence (21.3 %) compared to breast cancer cases from GWU (18.2 %) and WHI (15.2 %).

**Conclusions:**

The prevalence of first-degree breast cancer family history among IBC cases was lower compared to breast and ovarian cancer cases but higher than unaffected individuals. Our multiple-case inflammatory and non-inflammatory breast cancer families may reflect aggregation of common genetic and/or environmental factors predisposing to both types of breast cancer. Our findings that oral contraceptive use and regular alcohol consumption may be associated with IBC warrant further investigations.

## Background

Inflammatory breast cancer (IBC) is a rare form of breast cancer characterized by an early average age of diagnosis (52 years versus 57 years for non-inflammatory breast cancer), aggressive histopathologic features (at least stage IIB at diagnosis), and poor survival [1]. Clinically, IBC is defined by the American Joint Committee on Cancer (AJCC) as a disease characterized by redness, warmth and edema (peau d’orange) involving at least one-third of the breast [2]. IBC accounts for an estimated 1.0 %-5.0 % of all incident breast carcinomas and 8.0–10.0 % of breast cancer mortality [3–5]. Variable incidence trends of IBC have been reported in various geographic areas and among different ethnic/racial groups, with increasing trends reported in the United States [4–6] and decreasing trends in Tunisia [7].

The etiologic components of IBC are generally unknown and the contributions of hereditary versus environmental/life style factors remain subject of controversy in the literature. Case reports and case-case studies of IBC have reported associations with factors such as early age at first birth [8, 9], high body mass index (BMI) [10, 11], trauma [12], and longer duration of breast feeding (hypothesized to increase risk through an estrogen surge) [9]. Reports of IBC clusters [8, 13], seasonal variations in the risk of IBC [14], higher occurrence in rural versus urban settings [15], and declining incidence with improving socioeconomic status [7] further highlight the role of environmental factors in the etiology of IBC. On the other hand, reports of familial IBC, including in association with *BRCA* mutations [16] suggest a role for heredity. Furthermore, genetic influences play a role in susceptibility to other malignancies with a major environmental component, such as lung and colorectal cancers [17–20].

In order to investigate the importance of hereditary factors in the etiology of IBC, we compared patients with IBC to several carefully chosen comparison groups with known prevalence of first-degree breast cancer family history. These comparison groups consisted of population-based and hospital-based non-inflammatory breast cancer and ovarian cancer cases as well as healthy controls (with no personal history of breast or ovarian cancer). As a secondary objective, comparisons were also made with respect to selected environmental/lifestyle risk factors for breast cancer.

## Methods

### Subject ascertainment and secondary data requisition

Patients with IBC (*n* = 141) and with non-inflammatory breast cancer (*n* = 178) were ascertained through epidemiologic studies conducted at George Washington University (GWU) from 2002 to 2012 [11, 21]. All subjects were Caucasian and the same epidemiologic questionnaire asking about reproductive, medical and family history was collected on both groups of patients at the time of recruitment. The family history portion of the questionnaire asked about diagnosis of breast cancer among first-degree relatives of the index case. We subsequently validated the family history questions of this questionnaire by collecting pedigrees on 21 of 67 living IBC patients and by correlating the information from pedigrees and the questionnaires. We found 100 % agreement between the two modes (questionnaire versus pedigree) of collecting first-degree breast cancer family history information. We also came across several families with cases of both inflammatory and non-inflammatory breast cancer among the relatives.

We compared the IBC cases with breast cancer cases and controls recruited through two large population-based studies, which were available to us as secondary datasets. Raw data from the Cancer Genetic Markers of Susceptibility (CGEMS) [22, 23] and the Women’s Health Initiative (WHI) [24, 25] cohorts were downloaded from the National Institutes of Health (NIH), database for Genotypes and Phenotypes (dbGaP) after obtaining approvals from the corresponding data repository committees at the NIH/dbGaP. All CGEMS and WHI subjects in our study were Caucasian; additional information about these secondary datasets is provided below.

Post-menopausal breast cancer cases unselected for age or family history (*n* = 1145) along with appropriately-matched healthy controls with no personal history of breast cancer (*n* = 1142) were available from the CGEMS breast cancer study [dbGaP accession #6175-10, version phs000147/GRU] nested within the Nurses’ Health Study (NHS) cohort [22, 23]. The CGEMS breast cancer cases and controls were compared to IBC cases with respect to the prevalence of first-degree breast cancer family history. Information on environmental risk factors on the CGEMS breast cancer cases and controls was not made available to us as part of the secondary data, therefore, comparisons with CGEMS were limited to the family history data.

Another group of post-menopausal breast cancer cases (≥50 years of age) unselected for family history (*n* = 465) and healthy controls with no personal history of breast or ovarian cancer (*n* = 9317) was obtained from the Hormone Therapy Trials of the WHI cohort [dbGaP accession #11296-6 for version phs000200/HMB-IRB-NPU and #11295-7 for version phs000200/HMB-IRB] [24, 25]. WHI cases and controls were compared to IBC cases with respect to the prevalence of first-degree breast cancer family history and selected environmental risk factors for breast cancer.

Given that many genetic and environmental risk factors for breast cancer also predispose to ovarian cancer [26] and that first-degree family history of breast cancer is a significant risk factor for ovarian cancer as well [26], we conducted similar comparisons with subjects who had been ascertained through a case–control study of ovarian cancer conducted at the University of Toronto (UT) and available to us as secondary data. The UT study had ascertained Ashkenazi Jewish women with ovarian cancer (*n* = 260, unselected for age or family history) and appropriately-matched controls (*n* = 331, with no personal history of ovarian cancer) from several hospitals in North America [27]. Detailed epidemiologic and family history information was collected at the time of ascertainment through structured interviews, the majority of which were conducted by RM. The main objectives of the UT ovarian cancer study were to determine the role of genetic and environmental risk factors and to characterize the *BRCA* genes [27, 28]. Genetic analysis as part of the original UT study had identified 96 carriers of the three *BRCA* Ashkenazi Jewish founder mutations (51 *BRCA1* 185delAG, 15 *BRCA1* 5382insC, and 30 *BRCA2* 6174delT carriers) and 164 non-carriers [27, 28].

### Statistical analysis

IBC cases were compared to breast and ovarian cancer cases and healthy controls with respect to the prevalence of first-degree family history of breast cancer and selected environmental/lifestyle risk factors for breast cancer. The environmental/lifestyle variables were selected based on availability of information on each variable from the majority of datasets and included parity, body mass index (BMI), regular alcohol use (≥1 drink per day), and ever use of oral contraceptives. CGEMS breast cancer cases and controls were only used for comparison of family history since information about environmental/lifestyle variables was not made available to us as part of the secondary data on these subjects. Unadjusted Odds ratios and 95 % confidence intervals were calculated. For the primary objective of comparing first-degree breast cancer family history, adjusted analyses (controlling for age, parity, BMI, regular alcohol use and ever use of oral contraceptives) were also conducted for comparison of GWU IBC and non-IBC cases. All statistical analyses were conducted using SAS version 9.1 (SAS Institute, Cary, NC) and Epi Info™ (http://www.cdc.gov/epiinfo).

## Results

Average age of diagnosis for IBC cases (48.3 years) was lower than that of non-inflammatory breast cancer cases from GWU (51.8 years) and WHI (73.6 years) as well as ovarian cancer cases (56.1 years) (Table 1). Average age of diagnosis for CGEMS breast cancer cases was not available but the investigators reported all cases to be post-menopausal at diagnosis [22]. Average age at study entry for CGEMS subjects was estimated from ranges provided as part of the secondary data (Table 1). Older average ages of diagnosis for WHI breast cancer cases reflect the postmenopausal inclusion criteria and the prospective study design of the WHI trials.

**Table 1:** Hereditary and Selected Environmental Risk Factors among Inflammatory Breast Cancer Cases and Comparison Groups

	GWU IBC^a^ Cases	GWU BC^a^ Cases	UT OC^b^ Cases	OC Cases^c^ *BRCA*	OC Cases^d^ non-*BRCA*	UT^b^ Controls	WHI BC^e^ Cases	WHI^e^ Controls	CGEMS BC^f^ Cases	CGEMS^f^ Controls
	*n* = 141 (%)	*n* = 178 (%)^g^	*n* = 260 (%)	*n* = 96 (%)	*n* = 164 (%)	*n* = 331 (%)	*n* = 465 (%)^g^	*n* = 9317 (%)^g^	*n* = 1145 (%)	*n* = 1142 (%)
Average Age at Study Entry	50.3	53.8	59.2	57.9	60.1	52.2	67.0	67.0	65.4^h^	65.6^h^
Average Age at Diagnosis	48.3	51.8	56.1	54.6	57.1	NA	73.6	NA	Unavailable^i^	NA
Average Body Mass Index (Kg/m^2^)	28.9	26.5	24.5	24.3	24.5	24.9	30.2	28.8		
First-Degree Breast Cancer Family History										
Yes	24 (17.0)	43 (24.4)	63 (24.2)	33 (34.4)	30 (18.3)	37 (11.2)	74 (16.9)	1105 (12.6)	274 (23.9)	204 (17.9)
Parity (≥1 Pregnancy)										
Yes	114 (80.9)	130 (73.9)	233 (89.6)	89 (92.7)	144 (87.8)	286 (86.4)	418 (90.1)	8661 (93.1)		
Oral Contraceptive Use										
Yes	113 (80.1)	125 (71.0)	101 (38.8)	43 (44.8)	58 (35.4)	216 (65.3)	159 (34.2)	3089 (33.1)		
Regular Alcohol Use (≥1 drink per day)										
Yes	30 (21.3)	32 (18.2)	35 (13.5)	16 (16.7)	19 (11.6)	42 (12.7)	69 (15.2)	1184 (12.8)		

^a^ Inflammatory breast cancer (IBC) and non-inflammatory breast cancer (BC) cases recruited at George Washington University (GWU). All subjects recruited at GWU and included in this study were Caucasian. Subjects were recruited within an average of two years post diagnosis

^b^ Ovarian Cancer (OC) cases and controls obtained from hospital-based case–control study conducted at University of Toronto (UT). All subjects recruited through the UT study were Caucasian and Ashkenazi Jewish

^c^ Ovarian cancer cases with an Ashkenazi Jewish founder mutation in the *BRCA1* (185delAG and 5382insC) and *BRCA2* (6184delT) genes

^d^ Ovarian cancer cases who tested negative for the three Ashkenazi Jewish founder mutations in the *BRCA1* or *BRCA2* genes

^e^ Breast cancer (BC) cases and controls obtained from the Hormone Therapy Trials of the population-based Women’s Health Initiative (WHI) cohort. All WHI cases and controls included in our study were Caucasian and 50 years of age or above at study entry

^f^ Breast Cancer (BC) cases and controls obtained from the Cancer Genetic Markers of Susceptibility (CGEMS) study conducted among participants in the population-based Nurses’ Health Initiative (NHS) cohort. All subjects in CGEMS were Caucasian and post-menopausal at recruitment

^g^ Percentages for some variables do not correspond to the total number of cases and controls due to “missing” answers

^h^ Estimated from age ranges provided as part of the secondary data as follows: <54, 55–59, 60–64, 65–69, 70–74, and ≥75

^i^ Average age of diagnosis was unavailable but all cases were reported to be post-menopausal at diagnosis and controls were matched to cases based on several criteria including age at diagnosis

First-degree breast cancer family history was defined as the presence of at least one case of breast cancer among the mothers and/or sisters of the index cases. The prevalence of first-degree breast cancer family history among the IBC cases (17.0 %) was lower in comparison to the prevalence among the non-inflammatory breast cancer cases from GWU (24.4 %), breast cancer cases from CGEMS (23.9 %), and ovarian cancer cases irrespective of the *BRCA* mutation status (24.2 %) (Table 1). Comparisons revealed nearly similar frequency of first-degree family history of breast cancer among IBC cases compared to WHI breast cancer cases (16.9 %), *BRCA*-negative ovarian cancer cases (18.3 %), and CGEMS controls (17.9 %), and higher prevalence compared to UT-controls (11.2 %) and WHI controls (12.6 %) (Table 1). None of the comparisons involving family history reached statistical significance; adjusted analyses of GWU data produced similar odds ratios to unadjusted analyses. These results suggest a lower or similar prevalence of first-degree breast cancer family history among IBC cases compared to hospital-based and population-based breast and ovarian cancer cases but higher prevalence compared to unaffected individuals.

We identified several multiplex IBC families. The IBC index case in one family had a mother, a paternal aunt, and a paternal uncle with breast cancer. The disease was pre-menopausal in one relative and a paternal cousin was reported to have died from ovarian cancer at age 30. The index case in another family reported a sister and two aunts with breast cancer, including one with premenopausal disease. Another family was notable for reported pre- and peri-menopausal breast cancer in two sisters of the index case and a maternal relative diagnosed in her late 30’s. To our knowledge, these are among the first reported families with multiple cases of non-inflammatory breast cancer among first- and second-degree relatives of the IBC index case.

Body mass index (BMI) is considered a risk factor for both IBC [10] and for non-inflammatory breast cancer (post-menopausal only) [29]. BMI for IBC cases in our study (28.9 kg/m^2^) was slightly higher than the BMI of breast cancer cases from GWU (26.5 kg/m^2^) and of ovarian cancer cases and controls (24.5 kg/m^2^ and 24.9 kg/m^2^, respectively) (Table 1) but the comparisons did not reach statistical significance. WHI breast cancer cases had higher BMI (30.2 kg/m^2^) compared to all other groups including the IBC cases, but comparisons did not reach statistical significance.

Nulliparity is a risk factor for both non-inflammatory breast cancer [30] and ovarian cancer [26] but has not been reported in association with the risk of IBC. IBC cases in our study had a higher prevalence of parous women (80.9 %) compared to non-inflammatory breast cancer cases from GWU (73.9 %) but the comparison did not reach statistical significance. The prevalence of parous women among IBC cases was significantly lower than the prevalence among WHI breast cancer cases (OR = 0.46, 95 % CI: 0.27–0.81) and WHI controls (OR = 0.31, 95 % CI: 0.20–0.49) as well as the prevalence among ovarian cancer cases (OR = 0.49, 95 % CI: 0.26–0.91).

Long-term use (>10 years) of oral contraceptives has been associated with an increased risk for non-inflammatory breast cancer [31] and its short-term use is believed to reduce the risk of ovarian cancer [32, 33]. Oral contraceptive use has not been previously reported in association with the risk of IBC. Data on duration of oral contraceptive use was not available on some of the comparison groups in our study and hence we limited our comparisons to ever use of oral contraceptives. The prevalence of ever use of oral contraceptives among IBC cases (80.1 %) was significantly higher compared to WHI breast cancer cases (OR = 7.77, 95 % CI: 4.82–12.59) and controls (OR = 8.14, 95 % CI: 5.28–12.61). IBC cases also reported a significantly higher ever use of oral contraceptives compared to ovarian cancer cases (OR = 6.35, 95 % CI: 3.83–10.62) and controls (OR = 2.15, 95 % CI: 1.31–3.54) from UT. The higher prevalence of oral contraceptive use among IBC cases in comparison to non-inflammatory breast cancer cases from GWU was of borderline statistical significance (OR = 1.71, 95 % CI: 0.98–2.99).

Excess alcohol consumption (>35 g per day) is an established risk factor for non-inflammatory breast cancer [34–37] but has not been previously reported in association with the risk of IBC. In our study, IBC cases had significantly higher prevalence of regular alcohol use (≥1 drink per day) compared to WHI controls (OR = 1.84, 95 % CI: 1.20–2.82) and UT controls (OR = 1.86, 95 % CI: 1.07–3.22). Of note, the prevalence of regular alcohol use was higher among IBC cases (21.3 %) compared to non-inflammatory breast cancer cases from GWU (18.2 %) and from WHI (15.2 %) (Table 1) but the difference did not reach statistical significance. The association between alcohol intake and increased risk of IBC needs further investigation in future studies.

## Discussion

We undertook this study to assess the importance of heredity in the etiology of IBC because of the many reports emphasizing the strong impact of the environment on IBC [7–9, 11–15]. We compared the prevalence of breast cancer among first-degree relatives (i.e., mothers and sisters) between IBC cases and several comparison groups chosen to represent case and control groups with a range of high and low prevalence of first-degree breast cancer family history. Breast cancer and ovarian cancer patients are expected to have a high prevalence of first-degree breast cancer family history [38, 39] while healthy individuals with no personal history of breast or ovarian cancer are expected to have a lower prevalence [40]. Our comparisons revealed that IBC cases in our study had a slightly lower or similar prevalence of first-degree breast cancer family history compared to breast and ovarian cancer patients but a higher prevalence compared to unaffected controls.

Up to twenty percent of breast cancer cases are believed to be hereditary (characterized by multiple cases of breast and/or other cancers among relatives) and due to segregation of high-penetrance [i.e., relative risk (RR) = 20)] susceptibility genes in the affected families [41]. Ovarian cancer has the highest rate of hereditary incidence world-wide; current estimates put the percentage of hereditary cases at 20 % of all cases of this disease [42]. Besides age, a family history of breast and ovarian cancer is the most significant risk factor for ovarian cancer [42]. Mutations in the breast and ovarian cancer susceptibility genes, *BRCA1* and *BRCA2*, are responsible for the majority of hereditary cases of breast cancer [41] and ovarian cancer [42]. In addition to the genetic link between breast and ovarian cancers, there are several shared environmental risk factors which predispose to both conditions [26]. Therefore, ovarian cancer was considered a suitable case group for comparison with IBC.

The prevalence of breast cancer among first-degree relatives of both breast and ovarian cancer cases in our study was between 16.9 % and 24.4 %, which is within the range reported in the literature (i.e., 6.2 %-27.1 % for general breast and ovarian cancer cases irrespective of *BRCA* mutation status [27, 28, 43–45]). Thus, IBC cases had slightly lower prevalence of first-degree breast cancer family history than the breast and ovarian cancer cases (irrespective of *BRCA* carrier status) in our study but a slightly higher prevalence than general breast and ovarian cancer cases in some studies reported in the literature. A recent nested case–control study in the Breast Cancer Surveillance Consortium (BCSC) investigated the association between IBC risk and number of factors, including family history of breast cancer. This study also found a higher risk of IBC in association with first-degree family history of breast cancer [46]. An earlier study had reported a 20 % frequency of breast cancer family history among IBC cases [47].

The prevalence of first-degree breast cancer family history among UT controls (11.2 %) and WHI controls (12.6 %) was slightly higher than the range (4.8–7.3 %) of estimates reported in the literature for healthy women with no personal history of breast or ovarian cancer [43, 45]. The even higher estimates for CGEMS controls (17.9 %) may reflect a particular aspect of the study design or subject characteristics of the CGEMS genome-wide association study [22]. Nonetheless, the prevalence of first-degree breast cancer family history among the IBC cases was higher than the prevalence among healthy individuals from the UT and WHI studies as well as the prevalence estimates reported in the literature.

We identified several IBC cases with non-inflammatory breast cancer and other cancers among first- and second-degree relatives, as discussed in the previous section. Three families with multiple cases of IBC were also identified in our study; one index case had a daughter with IBC, one had a sister with IBC and the third had a cousin with IBC. A case report of hereditary IBC in a mother and a daughter had identified a deleterious *BRCA2* mutation responsible for the disease in that family [16]. Accurate estimates of *BRCA* mutation frequency among IBC cases from systematic studies remain to be reported. Our results indicate that a proportion of IBC cases occur in the context of familial breast cancer which may be explained by aggregation of common hereditary factors and/or shared environmental risk factors predisposing to both inflammatory and non-inflammatory breast cancer in these families. Our findings and the previous case report of hereditary IBC highlight the importance of obtaining pedigrees on IBC cases and noting all diagnoses of cancer and benign disease among the relatives.

As part of this study, we also conducted comparisons between IBC and other groups with respect to the prevalence of selected environmental/life style risk factors for breast cancer, such as parity, BMI, oral contraceptive use, and consumption of alcoholic beverages. These comparisons were secondary to the main study objective and the secondary nature of some of the comparison groups was a limitation with respect to the ability to conduct a comprehensive analysis of all relevant environmental/lifestyle factors. Therefore, our findings with respect to the selected environmental/life style factors need to be interpreted with caution. Our comparisons revealed a higher prevalence of nulliparity, oral contraceptive use and regular alcohol consumption (≥1 drink on daily basis) among IBC cases.

The prevalence of ever use of oral contraceptives was significantly higher among IBC cases (80.1 %) compared to all other comparison groups including breast cancer and ovarian cancer cases in our study. The differences between groups with respect to oral contraceptive use may be due to differences in age or other confounding factors between groups. Therefore, the association between oral contraceptive use and IBC risk needs further investigation in future studies accounting for all potential confounding variables. We had previously reported associations between aggressive (high-grade) breast cancer with long-term oral contraceptive use [11].

Our findings with respect to the association between alcohol consumption and IBC are also novel. The prevalence of regular alcohol use (≥1 drink per day) was higher among IBC cases compared to all other comparison groups in our study. These findings are particularly noteworthy given that excess alcohol consumption (>35 g per day) is an established risk factor for non-inflammatory breast cancer [34–37]. Ethanol and acetaldehyde have been classified as group I carcinogens (i.e., carcinogenic to humans) by International Agency for Research on Cancer (IARC) and are believed to be the carcinogenic components of alcohol [37]. IARC classification is based on sufficient evidence in humans for carcinogenicity of alcohol consumption as demonstrated from case–control, cohort and mechanistic studies [48]. Carcinogenic components of alcohol may exert their effects directly through inducing genetic or epigenetic mutations or indirectly through inducing inflammatory processes [48]. The role of inflammatory processes in IBC development is not known. A number of markers of inflammation such as NF-kB, Cox, and JAK/STAT signaling have been suggested to play a role in the tumorigenesis of IBC [49]. Inflammation may contribute to IBC development through promoting proliferation, creating a pro-inflammatory tumor microenvironment, and/or promoting metastasis of malignant cells [49].

As mentioned earlier, the secondary nature of some of the comparison groups is a limitation of our study, particularly with respect to analysis of environmental/life style factors. Strengths of our study include systematic data collection through the use of epidemiologic questionnaires on all case and control groups; the exact same questionnaire was used on the IBC and non-inflammatory breast cancer cases from GWU. Other strengths include validation of the family history-related questions through collection of detailed pedigrees on a subset of IBC cases and access to raw (secondary) data from several case–control studies, which could be used as additional comparison groups. The case–control comparison groups in our study were sufficiently large to allow statistically meaningful comparisons and the unaffected individuals used as controls in these studies were appropriately matched to the breast and ovarian cancer cases on important criteria such as age and ethnicity.

## Conclusion

In summary, our study found the prevalence of first-degree family history of breast cancer among IBC cases to be slightly lower or similar compared to breast and ovarian cancer cases but higher than unaffected individuals. Therefore, similar contribution of hereditary components to IBC risk as that to non-inflammatory breast cancer risk cannot be ruled out. Our findings of multiple-case inflammatory and non-inflammatory breast cancer families may further reflect aggregation of genetic factors and gene-environment interactions predisposing to both types of breast cancer. Our findings that oral contraceptive use and regular alcohol consumption may increase the risk of IBC are noteworthy due to modifiable nature of these potential risk factors. Collection of multi-generation pedigrees and detailed epidemiologic information noting the factors associated with an increased risk of IBC in studies by us and others will help decipher the etiologic role of hereditary and environmental factors in susceptibility to IBC in future studies. Because of the rarity of IBC and multiplex IBC families, the importance of developing consortia to enable epidemiologic investigations including gene-environment interaction studies should be emphasized.

## Abbreviations

AJCC, American Joint Committee on Cancer; BC, Breast Cancer; BMI, Body Mass Index; CGEMS, Cancer Genetic Markers of Susceptibility; GWU, George Washington University; IBC, Inflammatory Breast Cancer; NHS, Nurses’ Health Study; OC, Ovarian Cancer; UT, University of Toronto; WHI: Women’s Health Initiative.

## References

[1] Dawood S, Merajver SD, Viens P, Vermeulen PB, Swain SM, Buchholz TA, Dirix LY, Levine PH, Lucci A, Krishnamurthy. International expert panel on inflammatory breast cancer: consensus statement for standardized diagnosis and treatment. Ann Oncol. 2011;22(3):515–23. 10.1093/annonc/mdq345 PMC3105293 20603440

[2] Edge S, Byrd DR, Compton CC, Fritz AG, Greene FL, Trotti A (2010). AJCC Cancer Staging Manual.

[3] Levine PH, Veneroso C (2008). The epidemiology of inflammatory breast cancer. Semin Oncol.

[4] Anderson WF, Schairer C, Chen BE, Hance KW, Levine PH (2005). Epidemiology of inflammatory breast cancer (IBC). Breast Dis.

[5] Hance KW, Anderson WF, Devesa SS, Young HA, Levine PH (2005). Trends in inflammatory breast carcinoma incidence and survival: the surveillance, epidemiology, and end results program at the National Cancer Institute. J Natl Cancer Inst.

[6] Chang S, Parker SL, Pham T, Buzdar AU, Hursting SD (1998). Inflammatory breast carcinoma incidence and survival: the surveillance, epidemiology, and end results program of the National Cancer Institute, 1975–1992. Cancer.

[7] Boussen H, Bouzaiene H, Ben Hassouna J, Dhiab T, Khomsi F, Benna F, Gamoudi A, Mourali N, Hechiche M, Rahal K, et al. Inflammatory breast cancer in Tunisia: epidemiological and clinical trends. Cancer. 2010;116(11 Suppl):2730–5. 10.1002/cncr.25175 20503401

[8] Duke TJ, Jahed NC, Veneroso CC, Da Roza R, Johnson O, Hoffman D. A cluster of inflammatory breast cancer (IBC) in an office setting: additional evidence of the importance of environmental factors in IBC etiology. Oncol Rep. 2010;24(5):1277–84. 10.3892/or_00000983 20878121

[9] Le MG, Arriagada R, Bahi J, Pfeiffer F, Cammoun M, Tabbane F, Rubino C. Are risk factors for breast cancer similar in women with inflammatory breast cancer and in those with non-inflammatory breast cancer? Breast. 2006;15(3):355–62. 10.1016/j.breast.2005.08.018 16198566

[10] Chang S, Buzdar AU, Hursting SD (1998). Inflammatory breast cancer and body mass index. J Clin Oncol.

[11] Veneroso C, Siegel R, Levine PH (2008). Early age at first childbirth associated with advanced tumor grade in breast cancer. Cancer Detect Prev.

[12] Hashmi S, Zolfaghari L, Levine PH (2012). Does secondary inflammatory breast cancer represent post-surgical metastatic disease?. Cancers (Basel).

[13] Levine PH, Hashmi S, Minaei AA, Veneroso C (2014). Inflammatory breast cancer clusters: A hypothesis. World J Clin Oncol.

[14] Levine PH, Liu Y, Veneroso C, Hashmi S, Cristofanilli M (2016). Seasonal variation in inflammatory breast cancer. Int J Virol Stud Res.

[15] Mourali N, Muenz LR, Tabbane F, Belhassen S, Bahi J, Levine PH (1980). Epidemiologic features of rapidly progressing breast cancer in Tunisia. Cancer.

[16] Jimenez AM, Growney A, Behrens G, Corbridge C, Chapman DD, Usha L (2012). Hereditary inflammatory breast cancer associated with BRCA2 mutation: a rare disease presentation in mother and daughter. Clin Adv Hematol Oncol.

[17] Mota P, Silva HC, Soares MJ, Pego A, Loureiro M, Cordeiro CR, Regateiro FJ. Genetic polymorphisms of phase I and phase II metabolic enzymes as modulators of lung cancer susceptibility. J Cancer Res Clin Oncol. 2015;141(5):851–60. 10.1007/s00432-014-1868-z PMC11824119 25388590

[18] Tan XL, Moslehi R, Han W, Spivack SD (2009). Haplotype-tagging single nucleotide polymorphisms in the GSTP1 gene promoter and susceptibility to lung cancer. Cancer Detect Prev.

[19] Tu L, Yan B, Peng Z. Common genetic variants (rs4779584 and rs10318) at 15q13.3 contributes to colorectal adenoma and colorectal cancer susceptibility: evidence based on 22 studies. Mol Genet Genomics. 2015;290(3):901–12. 10.1007/s00438-014-0970-x 25475391

[20] Moslehi R, Chatterjee N, Church TR, Chen J, Yeager M, Weissfeld J, Hein DW, Hayes RB. Cigarette smoking, N-acetyltransferase genes and the risk of advanced colorectal adenoma. Pharmacogenomics. 2006;7(6):819–29. 10.2217/14622416.7.6.819 16981843

[21] Levine PH, Zolfaghari L, Young H, Hafi M, Cannon T, Ganesan C, Veneroso C, Brem R, Sherman M. What is inflammatory breast cancer? Revisiting the case definition. Cancers (Basel). 2010;2(1):143–52. 10.3390/cancers2010143 PMC3827596 24281037

[22] Hunter DJ, Kraft P, Jacobs KB, Cox DG, Yeager M, Hankinson SE, Wacholder S, Wang Z, Welch R, Hutchinson A, et al. A genome-wide association study identifies alleles in FGFR2 associated with risk of sporadic postmenopausal breast cancer. Nat Genet. 2007;39(7):870–4. 10.1038/ng2075 PMC3493132 17529973

[23] Haiman CA, Chen GK, Vachon CM, Canzian F, Dunning A, Millikan RC, Wang X, Ademuyiwa F, Ahmed S, Ambrosone CB, et al. A common variant at the TERT-CLPTM1L locus is associated with estrogen receptor-negative breast cancer. Nature genetics. 2011;43(12):1210–4. 10.1038/ng.985 PMC3279120 22037553

[24] Rossouw JE, Anderson GL, Prentice RL, LaCroix AZ, Kooperberg C, Stefanick ML, Jackson RD, Beresford SA, Howard BV, Johnson KC, et al. Risks and benefits of estrogen plus progestin in healthy postmenopausal women: principal results From the Women’s Health Initiative randomized controlled trial. JAMA. 2002;288(3):321–33. 10.1001/jama.288.3.321 12117397

[25] Anderson GL, Limacher M, Assaf AR, Bassford T, Beresford SA, Black H, Bonds D, Brunner R, Brzyski R, Caan B, et al. Effects of conjugated equine estrogen in postmenopausal women with hysterectomy: the Women's Health Initiative randomized controlled trial. JAMA. 2004;291(14):1701–12. 10.1001/jama.291.14.1701 15082697

[26] Zografos GC, Panou M, Panou N (2004). Common risk factors of breast and ovarian cancer: recent view. Int J Gynecol Cancer.

[27] Moslehi R, Chu W, Karlan B, Fishman D, Risch H, Fields A, Smotkin D, Ben-David Y, Rosenblatt J, Russo D, et al. BRCA1 and BRCA2 mutation analysis of 208 Ashkenazi Jewish women with ovarian cancer. Am J Hum Genet. 2000;66(4):1259–72. 10.1086/302853 PMC1288193 10739756

[28] Moslehi R, Singh R, Lessner L, Friedman JM (2010). Impact of BRCA mutations on female fertility and offspring sex ratio. Am J Hum Biol.

[29] van den Brandt PA, Spiegelman D, Yaun SS, Adami HO, Beeson L, Folsom AR, Fraser G, Goldbohm RA, Graham S, Kushi L, et al. Pooled analysis of prospective cohort studies on height, weight, and breast cancer risk. Am J Epidemiol. 2000;152(6):514–27. 10.1093/aje/152.6.514 10997541

[30] Kelsey JL, Gammon MD, John EM (1993). Reproductive factors and breast cancer. Epidemiol Rev.

[31] Collaborative Group on Hormonal Factors in Breast Cancer (1996). Breast cancer and hormonal contraceptives: collaborative reanalysis of individual data on 53 297 women with breast cancer and 100 239 women without breast cancer from 54 epidemiological studies. Lancet.

[32] Ferris JS, Daly MB, Buys SS, Genkinger JM, Liao Y, Terry MB (2014). Oral contraceptive and reproductive risk factors for ovarian cancer within sisters in the breast cancer family registry. Br J Cancer.

[33] Moorman PG, Havrilesky LJ, Gierisch JM, Coeytaux RR, Lowery WJ, Peragallo Urrutia R, Dinan M, McBroom AJ, Hasselblad V, Sanders GD, et al. Oral contraceptives and risk of ovarian cancer and breast cancer among high-risk women: a systematic review and meta-analysis. J Clin Oncol. 2013;31(33):4188–98. 10.1200/JCO.2013.48.9021 24145348

[34] Chen WY, Rosner B, Hankinson SE, Colditz GA, Willett WC (2011). Moderate alcohol consumption during adult life, drinking patterns, and breast cancer risk. JAMA.

[35] Tjonneland A, Christensen J, Olsen A, Stripp C, Thomsen BL, Overvad K, Peeters PH, van Gils CH, Bueno-de-Mesquita HB, Ocke MC, et al. Alcohol intake and breast cancer risk: the European Prospective Investigation into Cancer and Nutrition (EPIC). Cancer Causes Control. 2007;18(4):361–73. 10.1007/s10552-006-0112-9 17364225

[36] Hamajima N, Hirose K, Tajima K, Rohan T, Calle EE, Heath Jr CW, Coates RJ, Liff JM, Talamini R, Chantarakul N, et al. Alcohol, tobacco and breast cancer--collaborative reanalysis of individual data from 53 epidemiological studies, including 58,515 women with breast cancer and 95,067 women without the disease. Br J Cancer. 2002;87(11):1234–45. 10.1038/sj.bjc.6600596 PMC2562507 12439712

[37] Scoccianti C, Lauby-Secretan B, Bello PY, Chajes V, Romieu I (2014). Female breast cancer and alcohol consumption: a review of the literature. Am J Prev Med.

[38] Claus EB, Risch N, Thompson WD (1993). The calculation of breast cancer risk for women with a first degree family history of ovarian cancer. Breast Cancer Res Treat.

[39] Lynch HT, Snyder C, Casey MJ (2013). Hereditary ovarian and breast cancer: what have we learned?. Ann Oncol.

[40] Ingham SL, Warwick J, Buchan I, Sahin S, O'Hara C, Moran A, Howell A, Evans DG. Ovarian cancer among 8,005 women from a breast cancer family history clinic: no increased risk of invasive ovarian cancer in families testing negative for BRCA1 and BRCA2. J Med Genet. 2013;50(6):368–72. 10.1136/jmedgenet-2013-101607 23539753

[41] Couch FJ, Nathanson KL, Offit K (2014). Two decades after BRCA: setting paradigms in personalized cancer care and prevention. Science.

[42] Prat J, Ribe A, Gallardo A (2005). Hereditary ovarian cancer. Hum Pathol.

[43] Warner E, Foulkes W, Goodwin P, Meschino W, Blondal J, Paterson C, Ozcelik H, Goss P, Allingham-Hawkins D, Hamel N, et al. Prevalence and penetrance of BRCA1 and BRCA2 gene mutations in unselected Ashkenazi Jewish women with breast cancer. J Natl Cancer Inst. 1999;91(14):1241–7. 10.1093/jnci/91.14.1241 10413426

[44] Antoniou AC, Pharoah PD, McMullan G, Day NE, Stratton MR, Peto J, Ponder BJ, Easton DF. A comprehensive model for familial breast cancer incorporating BRCA1, BRCA2 and other genes. Br J Cancer. 2002;86(1):76–83. 10.1038/sj.bjc.6600008 PMC2746531 11857015

[45] Collaborative Group on Hormonal Factors in Breast Cancer (2001). Familial breast cancer: collaborative reanalysis of individual data from 52 epidemiological studies including 58,209 women with breast cancer and 101,986 women without the disease. Lancet.

[46] Schairer C, Li Y, Frawley P, Graubard BI, Wellman RD, Buist DS, Kerlikowske K, Onega TL, Anderson WF, Miglioretti DL. Risk factors for inflammatory breast cancer and other invasive breast cancers. J Natl Cancer Inst. 2013;105(18):1373–84. 10.1093/jnci/djt206 PMC3776263 24046390

[47] Aziz SA, Pervez S, Khan S, Kayani N, Azam SI, Rahbar MH (2001). Case control study of prognostic markers and disease outcome in inflammatory carcinoma breast: a unique clinical experience. Breast J.

[48] Dumitrescu RG, Shields PG (2005). The etiology of alcohol-induced breast cancer. Alcohol.

[49] Fouad TM, Kogawa T, Reuben JM, Ueno NT (2014). The role of inflammation in inflammatory breast cancer. Adv Exp Med Biol.

